# *SCR-22* of pollen-dominant *S* haplotype class is recessive to *SCR*-*44* of pollen-recessive *S* haplotype class in *Brassica rapa*

**DOI:** 10.1038/s41438-018-0103-5

**Published:** 2019-02-01

**Authors:** Chun-Lei Wang, Zhi-Ping Zhang, Eriko Oikawa, Hiroyasu Kitashiba, Takeshi Nishio

**Affiliations:** 1 School of Horticulture and Plant Protection, Joint International Research Laboratory of Agriculture and Agri-Product Safety, Key Laboratory of Plant Functional Genomics of the Ministry of Education, Yangzhou University, 48 Wenhui East Road, Yangzhou, 225009 People’s Republic of China; 2 Graduate School of Agricultural Science, Tohoku University, 468-1 Aza-Aoba, Aramaki, Aoba-ku, Sendai, Miyagi 980-0845 Japan

**Keywords:** Gene expression, Plant hybridization

## Abstract

*SCR/SP11* encodes the male determinant of recognition specificity of self-incompatibility (SI) in *Brassica* species and is sporophytically expressed in the anther tapetum. Based on dominance relationships in pollen and nucleotide sequence similarity, the *S* haplotypes in *Brassica* have been classified as class I or class II, with class-I *S* haplotypes being dominant over class-II *S* haplotypes. Here, we revealed that *S-22* in *B. rapa* belonging to class I is recessive to class-II *S-44* and class-I *S-36* in pollen, whereas it is dominant over *S-60*, *S-40*, and *S-29* based on pollination tests. *SCR/SP11* of *S-22* (*SCR-22*) was sequenced, revealing that the deduced amino-acid sequence of SCR-22 has the longest C-terminal domain among the SCR/SP11 sequences. The expression of *SCR-22* was found to be suppressed in *S-22/S-44* and *S-22/S-36* heterozygotes. Normal transcription of *SCR-44* was considered to be due to the transcription suppression of *Smi* sRNA of the *S-22* haplotype and a very low methylation state of the *SCR-44* promoter region in the tapetum of *S-22/S-44* heterozygotes. In *SCR-22*, only the cytosine residue located at the –37 bp position of the promoter region was hypermethylated in the tapetum of *S-22/S-44* heterozygotes, and few methylated cytosines were detected in the promoter and coding regions of *SCR-22* in *S-22/S-36* heterozygotes. *SCR-22* was also expressed in microspores in *S-22* homozygotes but not in *S-22/S-44* and *S-22/S-36* heterozygotes. These results suggest that a mechanism different from class-II *SCR*/*SP11* suppression may operate for the suppression of recessive class-I *SCR-22* in *S* heterozygotes.

## Self-incompatibility: Rethinking hierarchy

Studies in cabbage plants shed new light on self-incompatibility mechanisms to avoid self-fertilization. In Brassica, self-incompatibility is controlled by the expression of dominant and recessive genetic variants in the S-locus region. These variants encode proteins that mediate the rejection of self-pollen, but little is known about how the dominance relationship is established between them. Takeshi Nishio at Tohoku University in Japan and colleagues have examined the sequence, expression pattern and dominance relationships of the gene encoding the pollen-coat protein SCR-22. Despite sharing features with dominant SCR variants, SCR-22 can act in a recessive manner. Interestingly, unlike other recessive SCR variants, the suppression of SCR-22 expression does not depend on the addition of methyl groups that prevent transcription factor binding. This finding suggests the dominance hierarchy is governed by different mechanisms.

## Introduction

Self-incompatibility (SI) is a genetic mechanism exploited by many angiosperm species to prevent inbreeding and to promote outcrossing. In most species, SI is controlled by a single *S* locus with a large number of haplotypes. The SI response occurs when the *S* haplotype of the pollen is the same as that of the pistil. In *Brassica*, three genes located at the *S* locus have been characterized, namely, *S*-receptor kinase (*SRK*), a female determinant of recognition specificity;^1,2^ *S*-locus cysteine-rich protein/*S*-locus protein 11 (*SCR*/*SP11*), a male determinant;^3,4^ and *S*-locus glycoprotein (*SLG*), which is highly similar to the extracellular domain (*S*-domain) of *SRK*^5^.

SCR/SP11 is a small cysteine-rich protein with ca. 50 amino-acid residues with 8 conserved cysteine residues. The sequence of mature SCR/SP11 protein is highly polymorphic, with less than 50% amino-acid similarity among different *S* haplotypes within the same species^3,6–9^. In most SCR/SP11 variants, only a few amino acids are conserved, such as the eight cysteines, a glycine between the first and second cysteines, and an aromatic amino-acid residue between the third and fourth cysteines^3,6,7,10^. SCR/SP11 proteins are mainly produced in the anther tapetum and are then transferred to the surface of mature pollen^3,10–12^. Therefore, the SI phenotype in pollen is consistent with the dominant *S* haplotypes carried by *S* heterozygous plants.

Based on dominance relationships relative to the other alleles in *S*-heterozygous plants and the nucleotide sequence similarity of *S*-locus genes, the *S* haplotypes in *Brassica* are classified into two groups: the pollen-dominant *S* haplotypes termed class I and the pollen-recessive *S* haplotypes termed class II^13^. The class-I *S* haplotypes are always dominant over the class-II *S* haplotypes in pollen. In *Brassica rapa*, *SCR*/*SP11* sequences of four class-II members, namely, *S-44*, *S-60*, *S-40*, and *S-29*, have been identified, and a linear dominance relationship has been demonstrated among them^14,15^. The expression patterns of class-I and class-II *SCR/SP11* alleles are slightly different. Class-II *SCR*/*SP11* alleles are expressed only in the anther tapetum, whereas class-I *SCR*/*SP11* alleles are expressed not only in the tapetum but also in microspores^3,10,16^. Phylogenetic analysis has revealed that class-II *SCR*/*SP11* alleles form a distinct group separate from class-I *SCR*/*SP11* alleles^15^. The promoter sequences of *SCR/SP11* alleles show little similarity between the two classes, which results in an expression pattern difference between class-I and class-II *SCR*/*SP11* alleles.

In heterozygotes with class-I and class-II *S* haplotypes, the class-II *SCR*/*SP11* is not expressed, indicating that the dominance relationships are regulated at the messenger RNA level of *SCR*/*SP11* alleles^15^. It was found that class-I *SCR*/*SP11* alleles having promoter defects, which are not transcribed, also caused suppression of recessive class-II *SCR*/*SP11* alleles^17^. Subsequent studies demonstrated that the expression suppression of recessive class-II *SCR*/*SP11* alleles results from their tissue-specific methylation of promoter sequences in the tapetum^18^. Further analysis showed that a sequence with high similarity to the target methylated region lies in a region flanked by dominant *SLG* alleles named *SP11*-methylation-inducer (*Smi*). An *Smi* sequence was used as a template to form a 24-nucleotide small noncoding RNA (sRNA), which induced the methylation of the promoter of a recessive *SCR*/*SP11* allele and repressed its transcription^19^. Recently, *Smi2* has been identified in a class-II *S* haplotype sequence and has been shown to control the linear dominance hierarchy of the four class-II *SCR* alleles^20^.

*S-22* in *B. rapa* has been reported to be recessive to *S-24, S-26*, and *S-43*, belonging to class-I *S* haplotypes, in pollen in the same way class-II *S-60* is recessive to *S-24, S-26, S-28*, and *S-43*^21^. In the stigma, *S-22* has been revealed to be recessive to *S-28* in class-I *S* haplotypes. The nucleotide sequences of *SLG* and *SRK* of *S-22* have been determined and deposited in the DDBJ (AB054060 and AB054061, respectively). Comparison of the nucleotide sequences and deduced amino-acid sequences of these alleles with those of other *SLG* and *SRK* alleles has revealed that *S-22* belongs to the class-I *S* haplotypes^8^. Nucleotide sequences of *SCR/SP11* of *S-22* have not been reported. Since *S-22* is ranked the lowest in the dominance hierarchy of *S* haplotypes among class-I *S* haplotypes, this haplotype may have some unique characteristics. In the present study, we found that *S-22* was recessive to *S-44* in class-II haplotypes in pollen. We identified *SCR/SP11* of *S-22* (*SCR-22* hereafter), finding that it has a unique feature in deduced amino-acid sequences. The expression of *SCR-22* was suppressed in the *S-22/S-44* heterozygote, but the cytosine methylation pattern in the *SCR-22* promoter was different from that of recessive class-II *SCR* alleles. These results may help us to better understand the mechanism controlling dominance relationships among *SCR*/*SP11* alleles.

## Results

### Dominance relationships between *S-22* and other *S* haplotypes of *B. rapa* in pollen

We analyzed the dominance relationships in pollen between *S-8*, *S-22*, and class-II *S* haplotypes of *B. rapa* by pollination tests. The class-I allele *S-8* was used as a control to demonstrate the typical behavior of a class-I allele. Our preliminary experiments show that *S-8* is codominant with *S-22* and *S-36* in pollen (data not shown). The pollen of heterozygotes with *S-22* or *S-8* and one class-II *S* haplotype was applied to stigmas of *S-22*, *S-8*, or class-II *S* homozygotes. The results showed that the pollen of *S-22/S-44* heterozygotes can germinate and penetrate the stigmas of *S-22* homozygotes but cannot penetrate the stigmas of *S-44* homozygotes (Table 1). This result indicated that *S-22* is recessive to *S-44*, which is different from other class-I *S* haplotypes generally dominant over *S-44*. Further pollination tests showed that the pollen grains of *S-22/S-60*, *S-22/S-40*, and *S-22/S-29* heterozygotes were incompatible with the stigmas of *S-22* homozygotes, whereas they were compatible with the stigmas of *S-60*, *S-40*, and *S-29* homozygotes, respectively, indicating that *S-22* is dominant over *S-60*, *S-40*, and *S-29* in pollen. In addition, the pollen tubes of *S-22/S-36* heterozygotes, of which *S-36* is a class-I *S* haplotype, penetrated the stigmas of *S-22* homozygotes, whereas these pollen tubes did not penetrate the stigmas of *S-36* homozygotes, revealing that *S-22* is recessive to *S-36*. The pollen of heterozygotes with *S-8* and one class-II *S* haplotype was incompatible with the stigmas of *S-8* homozygotes, whereas they were compatible with the stigmas of *S-44*, *S-60*, *S-40*, and *S-29* homozygotes, indicating that *S-8* is dominant over all four class-II alleles in pollen.

**Table 1:** Reciprocal pollination tests between plants having different *S* genotypes in *B. rapa*

Stigma	Pollen								
	***S-22***/***S-36***	***S-22***/***S-44***	***S-22***/***S-60***	***S-22***/***S-40***	***S-22***/***S-29***	***S-8***/***S-44***	***S-8***/***S-60***	***S-8***/***S-40***	***S-8***/***S-29***
***S-22*** */* ***S-22***	+	+	–	–	–				
***S-8*** */* ***S-8***						–	–	–	-
***S-36*** */* ***S-36***	–								
*S-44/S-44*		–				+			
*S-60/S-60*			+				+		
*S-40/S-40*				+				+	
*S-29/S-29*					+				+

Compatible and incompatible pollination are shown by + and –, respectively. The class-I alleles are indicated in bold

### Sequence analysis of *SCR-22* of *B. rapa*

We determined the nucleotide sequence of the coding region of *SCR-22* and its promoter region sequence in two steps. Since *SCR-22* was not amplified by reverse transcription-polymerase chain reaction (RT-PCR) using the primers reported by Watanabe et al.^6^ and Sato et al.^8^, we amplified a partial sequence of *SCR-22* of *B. rapa* using many combinations of primers including newly designed primers (Supplementary Table S1). Second, we amplified the flanking sequence of the identified region of *SCR-22* by inverse PCR to determine the nucleotide sequence of the whole coding region of *SCR-22* and its promoter region. Our results showed that the coding region of *SCR-22* is 627 bp in length and contains a 306 bp intron (Supplementary Figure S1).

**Fig. 1:**
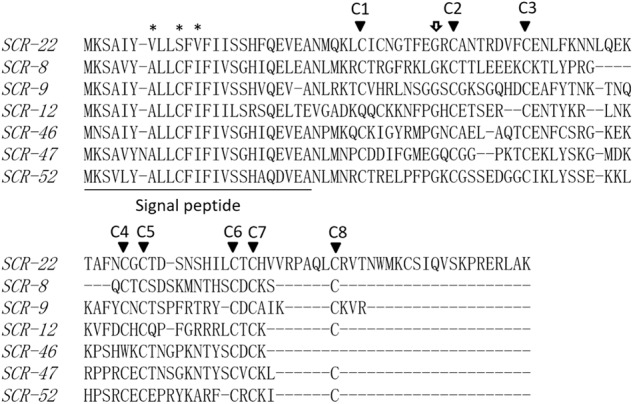
Alignment of the deduced amino-acid sequences of seven class-I SCR/SP11 proteins. Gaps were introduced to optimize the alignment. Asterisks indicate nonconserved amino acids in the putative signal peptides of SCR-22. Black arrowheads indicate the eight conserved cysteine residues. The white arrow indicates the conserved glycine residue

It was found that three amino acids, i.e., the seventh, tenth, and twelfth amino acids, in the putative signal peptide of SCR-22 are different from those of other class-I SCR sequences (Fig. 1). As with other SCR/SP11 proteins, the eight conserved cysteine residues are present in SCR-22, and a glycine residue between C1 and C2 is also conserved in SCR-22. However, the length of the SCR-22 protein is different from that of other SCR/SP11 proteins. Most SCR/SP11 proteins contain approximately 50 amino acids, whereas the SCR-22 protein contains approximately 70 amino acids. SCR-22 has a longer C-terminal domain (Fig. 1). Linkage analysis showed that this gene was linked to *SRK*-*22* in *B. rapa* (Supplementary Figure S2). These results confirm that the gene we identified is *SCR*-*22* of *B. rapa*.

### Tapetum isolation

**Fig. 2:**
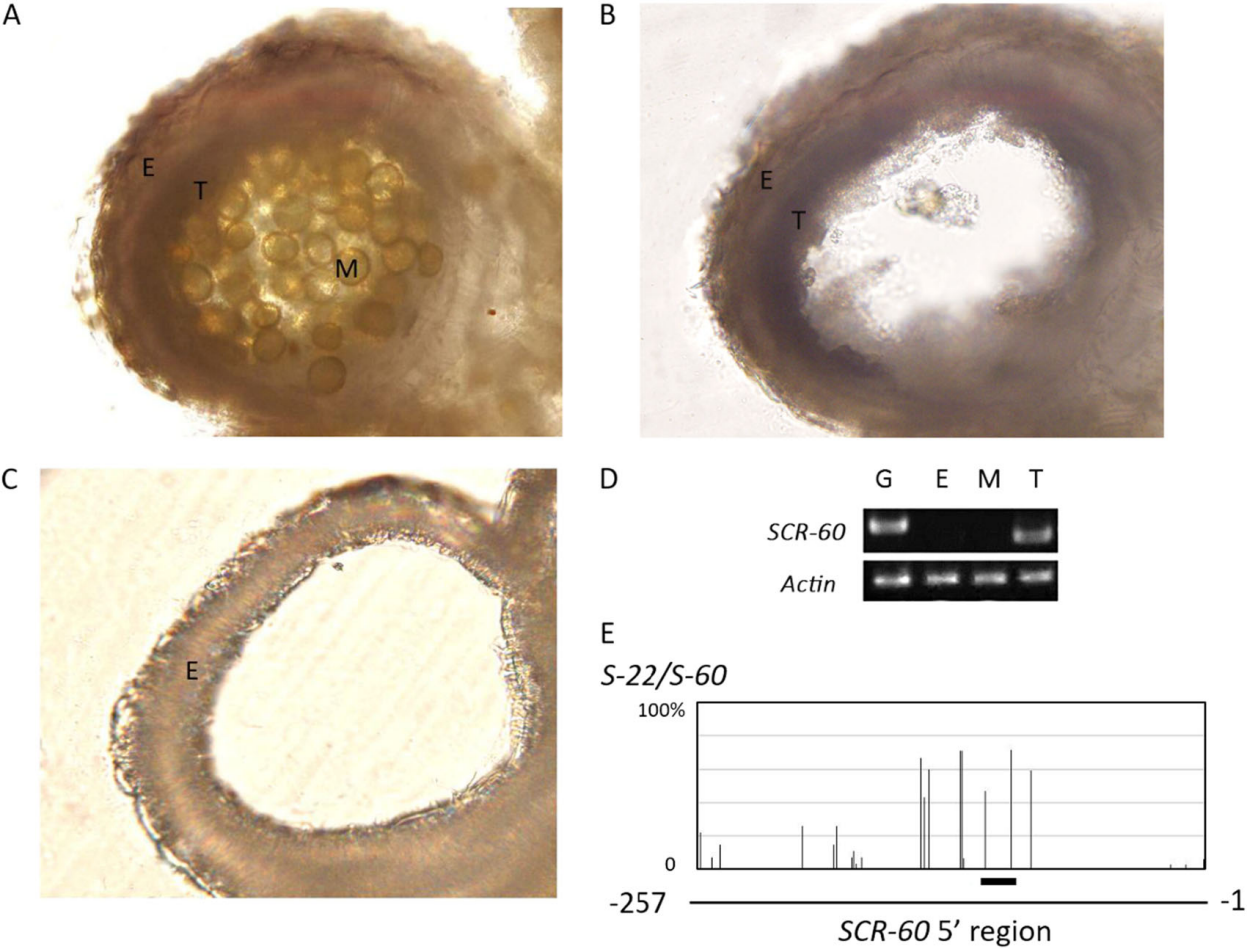
Validation of the method for tapetum isolation. Before the tapetum is isolated, an anther contains endothecium, tapetum, and microspores (**a**). Microspores were released by vortexing (**b**). After tapetum isolation, the tapetum and microspores were removed, and the endothecium had integrity (**c**). The expression of *SCR-60* was detected in the isolated tapetum of *S-60* homozygotes (**d**). The DNA methylation state of the *SCR-60* promoter in the tapetum of *S-22/S-60* heterozygotes was analyzed using the tapetum isolated by our method (**e**). The thick line under the DNA methylation profile indicates the region homologous to *Smi*. E endothecium, T tapetum, M microspores, G genomic DNA

It has been reported that class-II *SCR/SP11* alleles are specifically transcribed in the tapetum^15^ and that DNA methylation of the promoter of recessive *SCR/SP11* alleles in heterozygotes also occurs in the tapetum^18^. In previous studies, DNA was extracted from the nuclei of tapetum cells, but the method was not described in detail^18^. We therefore developed a method for tapetum isolation. The anther contains endothecium, tapetum, and microspores (Fig. 2a). We cut away both ends of the anthers and released the microspores by vortexing. All microspores were released (Fig. 2b). The anthers were then treated with cellulase RS and pectolyase Y23 for only 7 min to release the tapetum cells (Fig. 2c). We then used this method to isolate the tapetum cells from the anthers of *S-60* homozygotes and analyzed the expression of *SCR-60* in isolated endothecium, microspores, and tapetum (Fig. 2d). The expression of *SCR-60* was detected only in the isolated tapetum cells, not in isolated endothecium or microspores, confirming that the fraction we obtained was the tapetum and that few tapetum cells were present in the isolated endothecium fraction or microspore fraction. The tapetum cells from the anthers of *S-22/S-60* heterozygotes were then isolated to assess the methylation rate of the recessive *SCR-60* 5’ region. Methylated cytosine residues at CpG, CpNpG, and CpNpN sites were widespread, and the methylation frequencies of the two cytosine residues in the region of *SCR-60* homologous to the *Smi* of the *S* locus^19^ were 46.9% and 71.9%, respectively (Fig. 2e). The highest methylation frequency of cytosine residues in the 5’ region of class-II *SCR/SP11* alleles has been reported to be approximately 80%^18,19^. Considering that the DNA methylation of *SCR/SP11* alleles is detected only in the tapetum and that the highest methylation frequency detected in the present study was close to the highest reported methylation frequency, it can be inferred that the purity of the isolated tapetum fraction prepared using our method is comparable to that obtained by the method of Shiba et al.^18^ and that the isolated tapetum fraction of our study can be used for the following analyses.

### Gene expression of *SCR/SP11* alleles in *S* heterozygotes

**Fig. 3:**
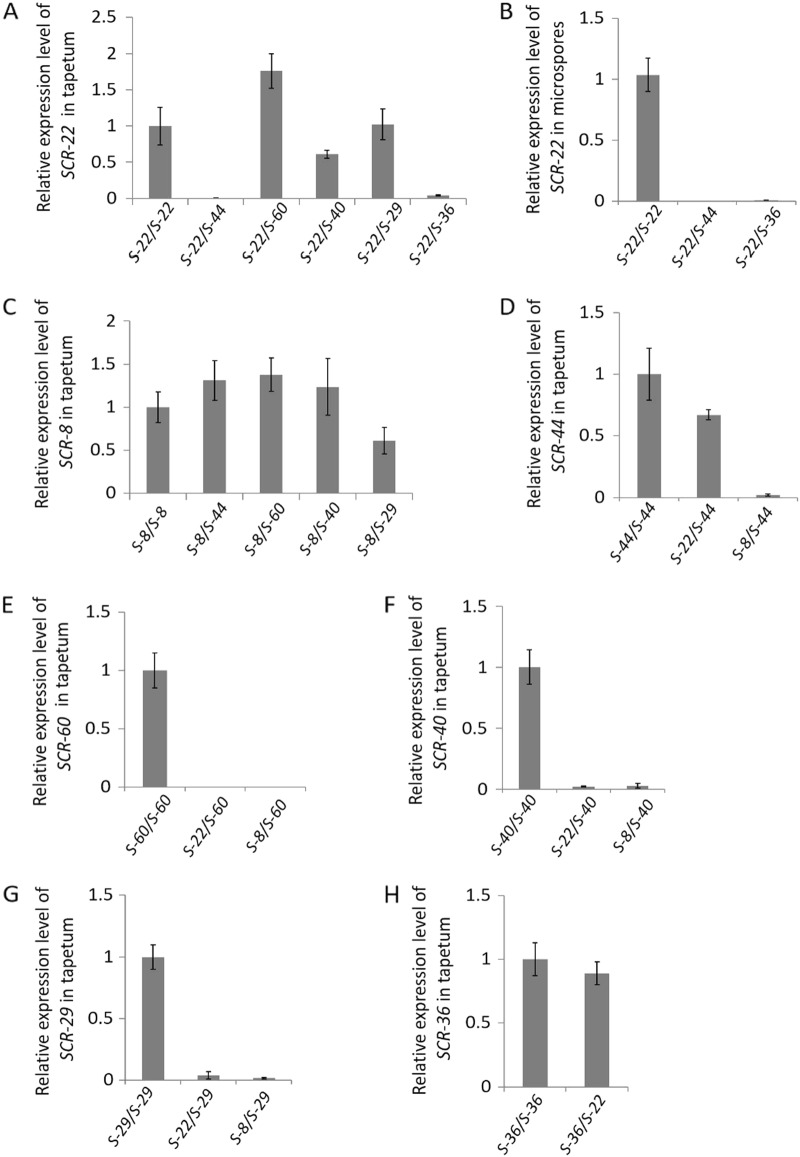
Relative expression levels of *SCR*/*SP11* alleles. The relative expression levels of *SCR-22* were detected in the tapetum of *S-22* homozygotes and *S-22*/*S-36*, *S-22*/*S-44*, *S-22*/*S-60*, *S-22*/*S-40*, and *S-22*/*S-29* heterozygotes (**a**) and in the microspores of *S-22* homozygotes and *S-22*/*S-36* and *S-22*/*S-44* heterozygotes (**b**); and those of *SCR-8* were detected in the tapetum cells of *S-8* homozygotes and *S-8*/*S-44*, *S-8*/*S-60*, *S-8*/*S-40*, and *S-8*/*S-29* heterozygotes (**c**). The relative expression levels of *SCR*-*44* were detected in the tapetum cells of *S-44* homozygotes and *S-22*/*S-44* and *S-8*/*S-44* heterozygotes (**d**), *SCR*-*60* levels were detected in *S-60* homozygotes and *S-22*/*S-60* and *S-8*/*S-60* heterozygotes (**e**), *SCR*-*40* levels were detected in *S-40* homozygotes and *S-22*/*S-40* and *S-8*/*S-40* heterozygotes (**f**), *SCR*-*29* levels were detected in *S-40* homozygotes and *S-22*/*S-29* and *S-8*/*S-29* heterozygotes (**g**), and *SCR*-*36* levels were detected in *S-36* homozygotes and *S-22*/*S-36* heterozygotes (**h**). Error bars represent standard errors (SE) of the mean of triplicate samples

The relative expression level of each *SCR/SP11* allele was investigated in the *S* heterozygotes. First, the expression level of *SCR-22* was measured by real-time quantitative PCR. The results showed that the expression of *SCR-22* in the tapetum cells of *S-22/S-60*, *S-22/S-40*, and *S-22/S-29* heterozygotes was the same as that in *S-22* homozygotes, whereas it was suppressed in the tapetum cells of *S-22/S-44* and *S-22/S-36* heterozygotes (Fig. 3a). *SCR-22* was expressed in the microspores of the *S-22* homozygotes, whereas the expression of *SCR-22* was suppressed in the microspores of the *S-22/S-44* and *S-22/S-36* heterozygotes (Fig. 3b). *SCR-8*, also belonging to class I, was expressed in the tapetum cells of all heterozygotes we analyzed (Fig. 3c). Second, the relative expression levels of four class-II *SCR/SP11* alleles were investigated. *SCR-44* was not expressed in the tapetum cells of *S-8/S-44* heterozygotes but was expressed in those of *S-22/S-44* heterozygotes (Fig. 3d). *SCR*-*60*, *SCR*-*40*, and *SCR*-*29* were not expressed in the tapetum cells of any of the *S* heterozygotes we analyzed (Fig. 3e, f, g). *SCR*-*36* was expressed in the tapetum cells of *S-22/S-36* heterozygotes (Fig. 3h). The observed relative expression levels suggested that *SCR*-*22* is dominant to *SCR*-*60*, *SCR*-*40*, and *SCR*-*29* and recessive to *SCR*-*44* and *SCR*-*36* and that *SCR*-*8* is dominant to all the class-II *SCR/SP11* alleles that we analyzed. These results were consistent with the results of the pollination tests.

### The methylation state of recessive *SCR* alleles in heterozygotes

**Fig. 4:**
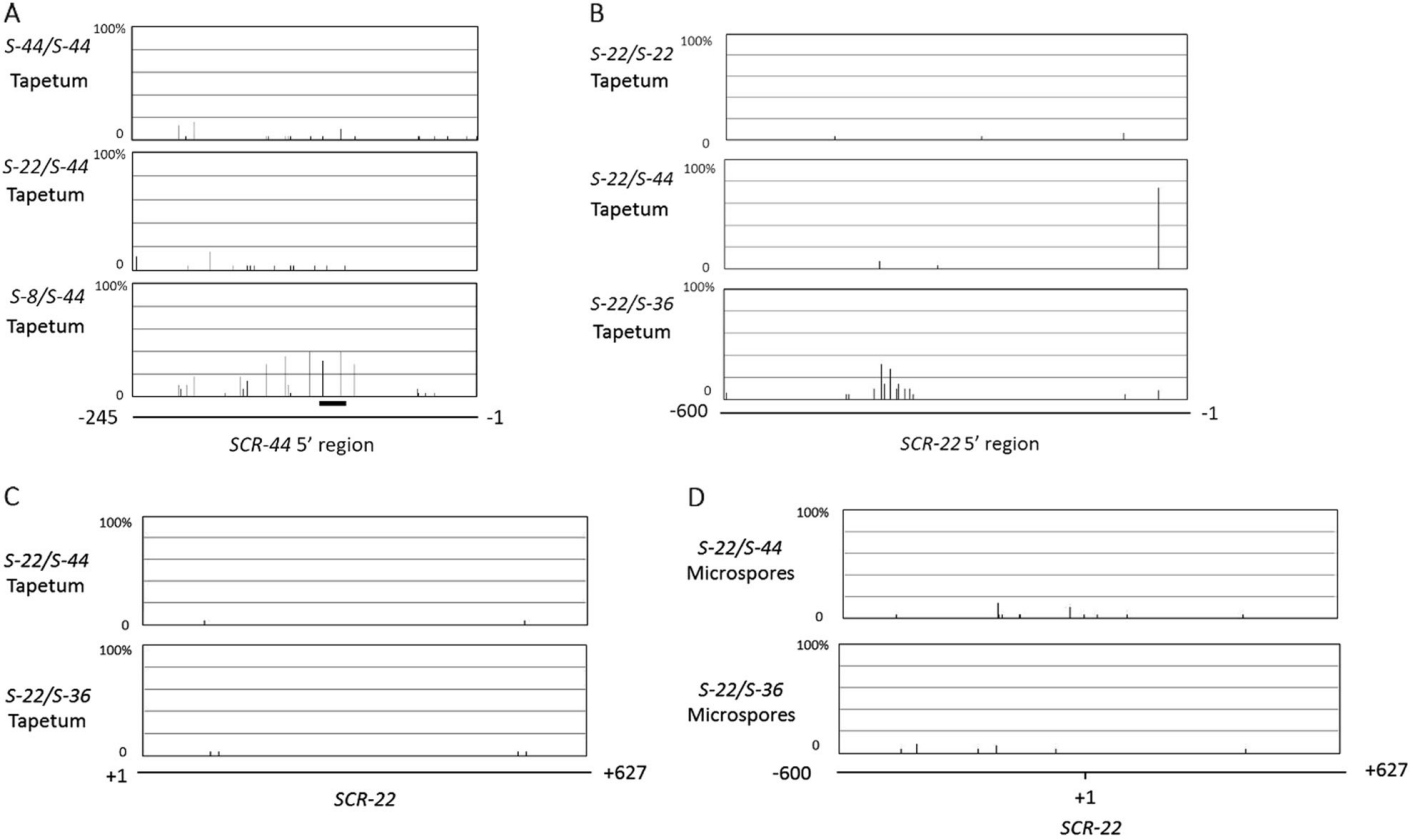
Analyses of the DNA methylation state of *SCR*-22 and *SCR*-*44*. Percentages of methylation at all cytosine residues in the *SCR-44* promoter (nucleotides –254 to –1) (**a**) and *SCR-22* promoter (nucleotides –600 to –1) (**b**) are shown in the histograms. **c** The DNA methylation state of the SCR-22 gene in the tapetum. **d** The DNA methylation state of the SCR-22 promoter and coding region in microspores. The results are from at least 30 cloned sequences. The thick line under the DNA methylation profile indicates the region homologous to *Smi*

It has been reported that suppression of the expression of recessive class-II *SCR/SP11* alleles results from methylation of the promoter region of recessive *SCR/SP11* alleles induced by an sRNA of the class-I or class-II *S* haplotype in the tapetum^19,20^. The methylation state of *SCR*-*44* was therefore measured in the present study. Widespread methylated cytosine residues were found in the *SCR*-*44* promoter region in the tapetum of *S-8/S-44* heterozygotes (Fig. 4a). All three types of cytosine methylation, i.e., CpG, CpNpG, and CpNpN, were detected in this region. The methylation frequencies of two cytosine residues in the region homologous to *Smi* in the *SCR-44* promoter of *S-8/S-44* heterozygotes were 32.1% and 39.3%, respectively. In the tapetum of *S-22/S-44* heterozygotes and *S-44* homozygotes, where *SCR*-*44* is transcribed, few methylated cytosine residues were detected in the promoter region of *SCR*-*44* (Fig. 4a). In addition, methylated cytosine residues in the region homologous to *Smi* in recessive *SCR*-*60*, *SCR-40*, or *SCR-29* were also observed, and the percentages of methylated cytosine were from 21.7% to 73.7% in the heterozygotes, which are higher than the 2.8% to 11.8% observed in the homozygotes (Supplementary Figure S3). These results indicate that the suppression of class-II *SCR/SP11* expression is related to DNA methylation.

The methylation states of *SCR*-*22* in *S-22* homozygotes and *S-22/S-44* and *S-22/S-36* heterozygotes were also investigated. In the tapetum of *S-22* homozygotes, the methylation rate of the *SCR*-*22* promoter region was very low (Fig. 4b). In the tapetum of *S-22/S-44* heterozygotes, where *SCR*-*22* was not transcribed, the cytosine residue located at the –37 position of the promoter was found to be highly methylated, with a methylation rate of 74% (Fig. 4b). No further methylated cytosine was detected in the *SCR*-*22* promoter region, coding region, and intronic region in the tapetum (Fig. 4b, c). At the same time, few methylated cytosines were detected in the *SCR*-*22* promoter region, coding region, and intronic region in the microspores of *S-22/S-44* heterozygotes (Fig. 4d), where *SCR*-*22* was also not transcribed. Additionally, in *S-22/S-36* heterozygotes, which carry two class-I *S* haplotypes, a methylated cytosine-rich region was detected at the promoter region (from –350 to –440) of *SCR*-*22* in the tapetum. However, the methylation rates of these cytosine residues were low, with the highest methylation rate being 31.2%, and only two types of cytosine methylation, i.e., CpNpG and CpNpN, were detected in this region. Few methylated cytosine residues were detected in the coding and intronic regions of recessive *SCR*-*22* (Fig. 4b–d). In the microspores of the *S-22/S-36* heterozygotes, similar to those of *S-22/S-44* heterozygotes, few methylated cytosines were detected in the *SCR*-*22* promoter region, coding region, and intronic region (Fig. 4b–d).

### Transcript analysis of *Smitrans*-acting sRNA

**Fig. 5:**
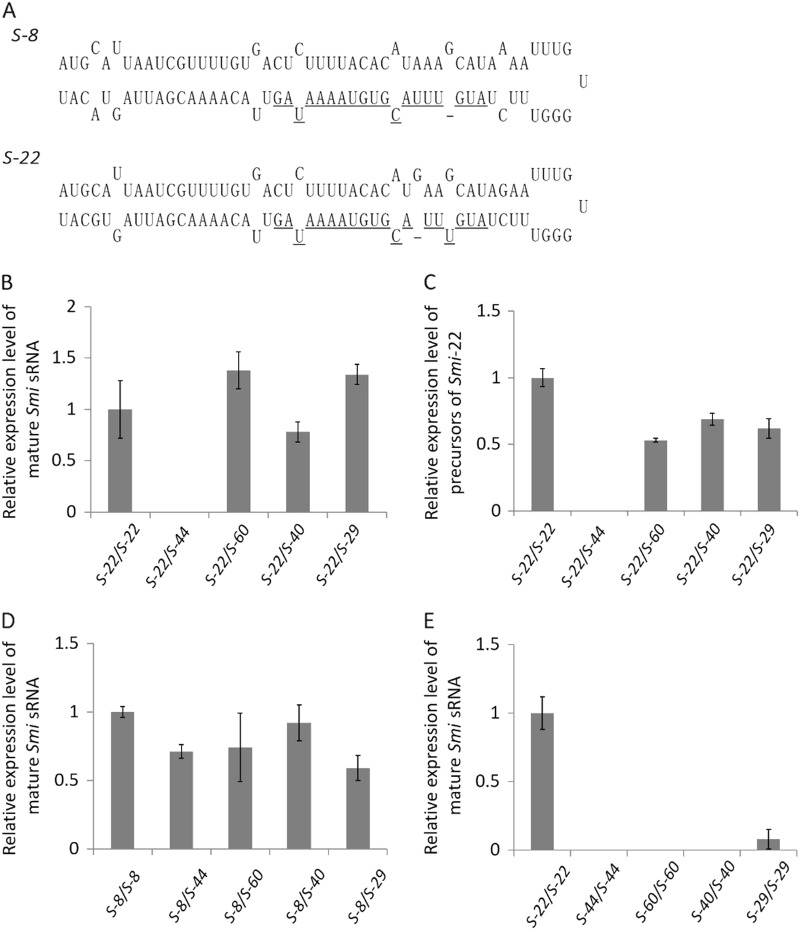
Relative expression levels of *Smi* sRNA. Stem-loop precursors predicted from *Smi-8* and *Smi-22* sequences are shown (**a**). The sequences of the mature sRNA are underlined. Mature *Smi* sRNA was detected by stem-loop RT-PCR in *S-22* homozygotes and heterozygotes with *S-22* and one class-II *S* haplotype (**b**). The transcription of precursors of *Smi-22* was also detected in *S-22* homozygotes and heterozygotes with *S-22* and one class-II *S* haplotype (**c**). Mature *Smi* sRNA in *S-8* homozygotes and heterozygotes with *S-8* and one class-II *S* haplotype (**d**) and in *S-22* homozygotes and class-II *S* haplotype homozygotes (**e**) was also detected. Error bars represent standard errors (SE) of the mean of triplicate samples

It has been reported that the *Smi* *trans*-acting sRNA from the class-I *S* locus induces the methylation of the promoter of recessive class-II *SCR/SP11* alleles^19^. The primer set SL-F1/SL-R1, designed by Tarutani et al.^19^, was used to amplify the sequence of the precursors of *Smi*-*8* and *Smi*-*22* from the *S* locus of *S-8* and *S-22* haplotypes. The results showed that the precursors can form an imperfect stem-loop structure (Fig. 5a), and the sequences of the *Smi*-*8* and *Smi*-*22* sRNAs are the same as that of the *Smi*-*9* sRNA^19^.

The mature sRNA expression levels of *Smi* in the tapetum cells of *S-22*, *S-8*, *S-44*, *S-60*, *S-40*, and *S-29* homozygotes and *S-22/S-44*, *S-22/S-60*, *S-22/S-40*, *S-22/S-29*, *S-8/S-44*, *S-8/S-60*, *S-8/S-40*, and *S-8/S-29* heterozygotes were analyzed by stem-loop RT-PCR. The results showed that mature sRNA of *Smi* is expressed in *S-22* homozygotes and *S-22/S-60, S-22/S-40*, and *S-22/S-29* heterozygotes but is totally suppressed in *S-22/S-44* heterozygotes (Fig. 5b). Furthermore, the expression of precursors of *Smi*-*22* was not detected in *S-22/S-44* heterozygotes (Fig. 5c). Mature sRNA of *Smi* was also detected in all plants having the *S-8* haplotype that we analyzed, including *S-8/S-44* heterozygotes (Fig. 5d). To confirm that the mature sRNA of *Smi* is formed from the class-I *S* locus, not from the class-II *S* locus, the expression level of mature sRNA of *Smi* was also analyzed in *S-44*, *S-60*, *S-40*, and *S-29* homozygotes. The expression of mature sRNA was hardly detected in the *S-29* homozygotes and was not detected in the *S-44*, *S-60*, and *S-40* homozygotes (Fig. 5e), confirming that the mature sRNA detected in the heterozygotes was mainly from the class-I *S* haplotypes. These results suggest that *SCR-44* expression in *S-22/S-44* heterozygotes is due to the suppression of *Smi* expression.

## Discussion

In the present study, we determined the nucleotide sequence of *SCR*-*22* of *B. rapa*. SCR-22 was found to be 20 amino acids longer than other SCR/SP11 proteins. Such a long SCR/SP11 protein has not previously been reported. In the putative signal peptide of SCR-22, three amino acids, i.e., the seventh, tenth, and twelfth, were not conserved. These changes are not considered to contribute to the difference in the hydrophobicity level of the SCR-22 signal peptide from that of other class-I SCR/SP11 proteins. The grand average of the hydropathicity value of the SCR-22 signal peptide was between those of SCR-8 and SCR-12, indicating that the function of the SCR-22 signal peptide was maintained.

*SCR-44* of class-II *SCR/SP11* alleles has been thought to be recessive to all class-I *SCR*/*SP11* alleles. In the present study, pollination tests showed that class-I *SCR-22* is recessive to *SCR-44* but dominant to *SCR*-*60*, *SCR*-*40*, and *SCR*-*29* (Table 1). The dominance relationships between *SCR-22* and class-II *SCR/SP11* alleles were further confirmed by gene expression analysis. The expression level of recessive *SCR*/*SP11* alleles is greatly reduced in *S* heterozygotes^15^. Our results showed that the expression of *SCR-22* is suppressed and that *SCR-44* is normally expressed in the tapetum of *S-22/S-44* heterozygotes (Fig. 2). Since the suppression of recessive class-II *SCR*/*SP11* transcription is considered to result from methylation of the promoter region induced by *Smi*, which can be observed in the tapetum^19,20^, we developed a method for tapetum isolation. Cytosine methylation was detected at a level comparable to that reported previously;^18,19^ therefore, our tapetum isolation method was found to be usable for analyses of the methylation states of the *SCR-22* promoter and the expression level of *Smi*.

Our investigation of dominance relationships showed that *SCR-44* was recessive to class-I *SCR-8* but dominant to class-I *SCR-22*. Methylation state analysis showed that the widespread methylated cytosine residues were present in the *SCR*-*44* promoter in *S-8/S-44* heterozygotes (Fig. 4). The methylation profile of the *SCR*-*44* promoter in *S-8/S-44* heterozygotes is similar to that of the *SCR*-*60* promoter in *S-52/S-60* heterozygotes^18,19^. The methylated cytosine residues of the *SCR*-*44* promoter at CpG, CpNpG, and CpNpN sites suggest that *Smi* sRNA triggers monoallelic de novo methylation in the recessive *SCR*-*44* promoter^19,22^. The methylation percentage of the *SCR*-*44* promoter in *S-8/S-44* was clearly lower than that of the *SCR*-*60* promoter in *S-52/S-60* heterozygotes and higher than the *SCR*-*60* promoter in *S-44/S-60* heterozygotes^18,19^. The methylation frequencies of two cytosine residues in the region homologous to *Smi* in the *SCR-44* of *S-8/S-44* heterozygotes were 32.1% and 39.3%, respectively. Methylated cytosine was also detected in the region homologous to *Smi* in the recessive *SCR*-*60*, *SCR*-*40*, or *SCR*-*29* in the heterozygotes (Supplementary Figure S3). In the homozygotes with class-II *SCR/SP11* alleles and *S-22/S-44* heterozygotes, few methylated cytosines were observed in the promoter of class-II *SCR/SP11* alleles. These results confirmed the suppression of recessive class-II *SCR/SP11* alleles induced by DNA methylation in their promoter regions.

Because *Smi* has been revealed to play a key role in inducing the promoter DNA methylation of recessive class-II *SCR/SP11* alleles, the precursor sequence of *Smi* from *S-8* and *S-22* was identified (Fig. 5a). The sequences of the mature *Smi*-*8* and *Smi*-*22* sRNAs were the same as that of the *Smi*-*9* sRNA^19^. The mature *Smi* sRNA was detected in the tapetum cells of *S-8* or *S-22* homozygotes and *S-8/S-44*, *S-8/S-60*, *S-8/S-40*, *S-8/S-29*, *S-22/S-60*, *S-22/S-40*, and *S-22/S-29* heterozygotes. However, mature *Smi* sRNA could not be detected in *S-22/S-44* heterozygotes. At the same time, class-I mature *Smi* sRNA could not be detected in the tapetum cells of *S-44*, *S-60*, or *S-40* homozygotes and was hardly detected in *S-29* homozygotes. These results confirm that the *Smi* sRNA detected in the present study was mainly from *S-8* or *S-22*. In addition, the precursors of *Smi*-*22* were not detected in *S-22/S-44* heterozygotes (Fig. 5c). Thus, the absence of *Smi* sRNA in *S-22/S-44* heterozygotes is considered to be due to the transcription suppression of *Smi*-*22* rather than a failure to cleave precursors of *Smi*-*22* into mature sRNA. In addition, in *S-22/S-44* heterozygotes, few methylated cytosines were detected in the promoter of *SCR-44*, indicating that the normal transcription of *SCR-44* resulted from the absence of *Smi* sRNA of *S-22*. Although *Smi* sRNA is not transcribed in *S-22*/*S-44* heterozygotes, it is transcribed in *S-22* homozygotes and in *S-22/S-60*, *S-22/S-40*, and *S-22/S-29* heterozygotes. The suppression of *Smi*-*22* transcription may be related to the function of the *S-44* haplotype. Most conserved microRNA (miRNA) genes are independent transcription units and have their own promoters^23^. Stress-responsive elements and tissue-specific regulatory elements have been found in the promoters of miRNA genes^24,25^. *Smi* is expressed in the tapetum specifically^19^, indicating that tissue-specific regulatory elements are present in the promoter region of the *Smi-22* gene. Transcription of *Smi-22* may be suppressed by some factor present in the *S*-locus sequence of *S-44*. The sequence of more than 10 kb of the *S-44* haplotype has been determined and published^26^, but it is not available in the sequence database. Therefore, repeated sequencing analysis is required for identification of the factor responsible for the suppression of *SCR-22* transcription.

Recently, *Smi2* was identified to control the linear dominance hierarchy of the four class-II *SCR* alleles^20^. It is possible that *SCR-22* expression is suppressed by *Smi2* of the *S-44* haplotype. However, no region similar to *Smi2* was found in the promoter region of *SCR-22*, indicating that the suppression of *SCR-22* expression in *S-22*/*S-44* heterozygotes was not related to the *Smi2* of *SCR*-*44*.

To examine whether the same suppression mechanism as that for class-II *SCR/SP11* participates in the suppression of recessive class-I *SCR-22* in *S* heterozygotes, the methylation state of recessive *SCR-22* in the tapetum was analyzed. The cytosine residue located at the –37 position of the *SCR-22* 5’ region was highly methylated in *S-22*/*S-44* heterozygotes (Fig. 4). In recessive class-II *SCR* alleles, methylated cytosine residues are widespread in the promoter region, with all three types of cytosine methylation, i.e., CpG, CpNpG, and CpNpN, occurring in the region^18,19^. However, in recessive class-I *SCR-22* in the tapetum of *S-22/S-44* heterozygotes, methylated cytosine was restrictedly localized at the –37 cytosine, and only CpNpN methylation was observed. In *S-22/S-36* heterozygotes, low levels of CpNpG and CpNpN methylation were detected in the region from –350 to –440  bp of the *SCR-22* 5’ region. Promoter hypermethylation around *cis*-regulatory elements could affect transcription repression by interfering with the transcription machinery^27,28^. It has been reported that the region around –192 bp of *SCR-9* contains the elements required for expression in the tapetum^12^. Alignment revealed that the 5’ region between –1 and –200 bp of *SCR-22* and *SCR-9* was highly conserved (Supplementary Figure S4), with the two sequences sharing 82.7% identity. Thus, the region around –191 bp of *SCR-22* is inferred to contain the elements required for expression in the tapetum. Our results showed that the hypermethylated cytosine located at the –37 position of the *SCR-22* 5’ region of the *S-22/S-44* heterozygotes or the –350 to –440 region of the *SCR-22* 5’ region with a low methylation rate in *S-22/S-36* heterozygotes is far from the core region for expression in the tapetum. Although a putative core binding sequence, CA(A/C)G(T/C)(T/C/A)(T/C/A), for a class of plant-specific NAC transcription factors was suggested to be present within the 5’ region (nucleotides –37 to –31) of *SCR-22* by a survey of putative *cis*-regulatory elements in silico^29^ (Supplementary Figure S4), this putative *cis*-regulatory element was not found within the promoter region of *SCR-47*. Thus, the binding sequence of these NAC transcription factors is not essential for the expression of all class-I *SCR/SP11* alleles in the tapetum. In addition, no methylated cytosine was detected in the class-I *SCR-22* in microspores of *S-22/S-44* or *S-22/S-36* heterozygotes, suggesting that the suppression of *SCR-22* expression is not induced by DNA methylation. Thus, the recessive *SCR-22* is considered to be suppressed through a mechanism without a DNA methylation pathway in general. The hypermethylated cytosine in the *SCR-22* promoter in the tapetum of *S-22/S-44* heterozygotes or the cytosine with a low methylation rate in the –350 to –440 region of the *SCR-22* 5’ region in the tapetum of *S-22/S-36* heterozygotes may be the result of histone modification^22^. These findings suggest that the suppression of transcription of *SCR-22* is not caused by the DNA methylation-mediated suppression through preventing the transcription factors from binding to their target sequence but possibly by the alteration of chromatin structure^22^. Therefore, a suppression mechanism different from that for class-II *SCR/SP11* may function in the suppression of recessive class-I *SCR-22* in *S* heterozygotes.

Class-I *SCR/SP11* alleles have been reported to be transcribed in both the tapetum and microspores^7,10,16^. The transcription of class-I *SCR/SP11* alleles in microspores occurs slightly later than that in the tapetum^12^. We found that *SCR-22* was expressed in microspores of *S-22* homozygotes but not in those of *S-22/S-44* and *S-22/S-36* heterozygotes. Although *S-44* and *S-36* were not present in the microspores having *S-22* in the *S-22/S-44* and *S-22/S-36* heterozygotes, the expression of *SCR-22* was suppressed, indicating that suppression of *SCR-22* expression may have been induced to occur before meiosis by a mechanism different from that caused by *Smi*. Further analyses are required to elucidate the suppression mechanism of recessive class-I *SCR/SP11* alleles in *S* heterozygotes.

## Methods

### Plant materials

*S-8*, *S-22*, *S-36*, *S-44*, *S-60*, *S-40*, and *S-29* homozygotes of *B. rapa*^2,30^ were used as the plant materials. Heterozygotes were obtained by cross-pollination of the *S* homozygotes.

### Pollination tests

Pollinated flowers were placed on solid agar for 24 h at 21 °C. Pistils were softened in 1 N NaOH at 55 °C for 1 h. The pistils were then stained with 0.1% aniline blue in 0.1 M K_3_PO_4_ and mounted in 60% glycerol. Pollen tubes were observed under a fluorescence microscope. Three flowers were used for each pollination, and the tests were replicated three times on different days.

### Amplification of *SCR-22* from *B. rapa*

Total RNA was extracted from anthers of *S-22* homozygotes using TRIzol reagent (Invitrogen, Shanghai, China). RNA was reverse-transcribed using a SuperScript™ III First-Strand Synthesis System (Invitrogen, Shanghai, China). A partial sequence of *SCR-22* was amplified by nested PCR using the primers SP11-1^31^, SP11-F1^6^, SP11-Fa, and SP11-1F8 as forward primers and Not1-(dT)18 as a reverse primer for the first PCR and SP11-2^31^, SP11-F2^6^, and SP11-2Fa as forward primers and RT1-long^9^ as a reverse primer for the second PCR. When SP11-1 and SP11-F2 were used for the first and second PCRs, a partial fragment of *SCR-22* was amplified. The promoter region and entire DNA sequence of *SCR-22* were identified by inverse PCR^32^. The primer sequences are listed in Supplementary Table S1.

### Tapetum isolation

Thirty flowers were collected 3 days before anthesis for collecting anthers. Both ends of the anthers were cut away, and the remaining part of the anthers was cut into two equal sizes. The two pieces were then placed into 1 mL tapetum isolation buffer (50 mM Hepes buffer, 0.5 M sucrose, and KOH to adjust the pH value to 7.5) in 1.5 mL centrifuge tubes. The tube was vortexed for 30 min to release microspores. The solution containing microspores was removed by filtration using nylon net (0.5 mm pore size) and washed three times with tapetum isolation buffer. The solution was centrifuged at 100 × *g* for 10 min, and the pellet contained microspores. Then, 1 mL isolation buffer (5% cellulase RS and 1% pectolyase Y23 in tapetum cell isolation buffer) was added to the anthers and vortexed for approximately 7 min. The upper solution was transferred to a new tube by filtration using a nylon net (0.5 mm pore size), and the upper solution was centrifuged at 20,000 × *g* for 10 min. The pellet contained the tapetum. Total RNA and DNA were isolated from the tapetum using TRIzol reagent (Invitrogen, Shanghai, China) for the following experiments.

To examine the adequacy of our method, the anthers collected before and after the isolation process were embedded in 5% agar. The anthers were sliced into 30 μM-thin sections by a DTK-3000 microslicer (Dosaka, Kyoto, Japan), and the sections were observed by a microscope.

### *SCR*/*SP11* expression analysis

Total RNA was isolated from the tapetum cells of *S-8*, *S-22*, *S-36*, *S-44*, *S-60*, *S-40*, and *S-29* homozygotes and *S-22*/*S-36*, *S-22*/*S-44*, *S-22*/*S-60*, *S-22*/*S-40*, *S-22*/*S-29*, *S-8*/*S-44*, *S-8*/*S-60*, *S-8*/*S-40*, and *S-8*/*S-29* heterozygotes. RNA was reverse-transcribed by the SuperScript™ III First-Strand Synthesis System (Invitrogen, Shanghai, China). Real-time RT-PCR was performed using SsoAdvanced^TM^ SYBR^®^ Green Supermix (Bio-Rad, Shanghai, China) on a Bio-Rad^®^ CFX96 system, following the manufacturer’s instructions. Each *SCR*/*SP11* region was amplified with specific primers (Supplementary Table S1). The *Actin* gene was used as an endogenous reference gene. The primers were confirmed to be approximately 90% to 100% efficient for amplification, and the 2^−∆∆CT^ method^33^ was used for all analyses. All reactions were performed in triplicate, and an average value was calculated for each set of reactions.

### DNA methylation state detection

DNA was isolated from the tapeta of *S-22* and *S-44* homozygotes and *S-22*/*S-36*, *S-22*/*S-44*, and *S-8*/*S-44* heterozygotes. The DNA was bisulfite treated with a MethylCode™ Bisulfite Conversion Kit (Applied Biosystems, Shanghai, China). The *SCR-44* promoter region and the promoter region, coding region, and intronic region of *SCR*-*22* modified by bisulfite were amplified using specific primers (Supplementary Table S1). Amplified PCR products were cloned into pGEM-T Easy vectors (Promega, Beijing, China), and at least 30 clones were sequenced.

### Detection of mature and precursor *Smi* sRNA

Detection of mature *Smi* sRNA was performed as previously described^34^. When microspores were in the uninucleate stage, small RNA was isolated from the anthers of *S-8*, *S-22*, *S-44*, *S-60*, *S-40*, and *S-29* homozygotes and *S-22*/*S-44*, *S-22*/*S-60*, *S-22*/*S-40*, *S-22*/*S-29*, *S-8*/*S-44*, *S-8*/*S-60*, *S-8*/*S-40*, and *S-8*/*S-29* heterozygotes using a *mir*Vana™ miRNA Isolation Kit (Ambion, Shanghai, China) and reverse-transcribed using SuperScript™ III RT with RT primers and U6-specific primers (Supplementary Table S1). The transcribed products were quantified using SsoAdvanced^TM^ SYBR^®^ Green Supermix (Bio-Rad, Shanghai, China) with small RNA-specific primers and universal primers (Supplementary Table S1). *U6* was used as an endogenous reference gene. The 2^−∆∆CT^ method^33^ was used for all analyses. All reactions were performed in triplicate, and an average value was calculated for each set of reactions.

For detection of precursor *Smi* sRNA, total RNA was isolated from anthers when microspores were at the uninucleate stage. RNA was reverse-transcribed by the SuperScript™ III First-Strand Synthesis System (Invitrogen, Shanghai, China) with precursor *Smi-22*-specific primers and Actin-R (Supplementary Table S1). Real-time RT-PCR was performed using SsoAdvanced^TM^ SYBR^®^ Green Supermix (Bio-Rad, Shanghai, China) on a Bio-Rad^®^ CFX96 system, following the manufacturer’s instructions. The *Actin* gene was used as an endogenous reference gene. The primers were confirmed to be approximately 90% to 100% efficient for amplification, and the 2^−∆∆CT^ method^33^ was used for all analyses.

### Accession numbers

The sequence data of *SCR-22*, *Smi-8*, and *Smi-22* were deposited in the GenBank of the National Center for Biotechnology Information (NCBI). The accession numbers are *Smi-8*, MG708355; *Smi-22*, MG708356; and *SCR-22*, MG708357.

## Electronic supplementary material

SUPPLEMENTAl MATERIAL
