# Supplementary material for: SCR-22 of pollen-dominant S haplotype class is recessive to SCR-44 of pollen-recessive S haplotype class in Brassica rapa
Source: Hortic Res. 2019 Feb 1;6:25. doi: 10.1038/s41438-018-0103-5 (PMC6355930; doi:10.1038/s41438-018-0103-5)
Supplement: Supplementary file 1 — SUPPLEMENTAl MATERIAL [file 41438_2018_103_MOESM1_ESM.docx]

**SUPPLEMENTAL MATERIAL**

**Supplemental Method**

**DNA methylation state detection by PCR-ELISA**

DNA was isolated from the tapetum fraction of *S-44*, *S-60*, *S-40*, and *S-29* homozygotes and *S-22*/*S-44*, *S-22*/*S-60*, *S-22*/*S-40*, *S-22*/*S-29*, *S-8*/*S-44*, *S-8*/*S-60*, *S-8*/*S-40*, and *S-8*/*S-29* heterozygotes. The DNA was bisulfite-treated with a MethylCode™ Bisulfite Conversion Kit (Applied Biosystems). The *SCR-44*, *SCR-60*, *SCR-40*, and *SCR-29* promoter regions modified by bisulfite were amplified using specific primers (Table S1). The forward primers were labelled with biotin at the 5’ end. Aliquot 5 µL PCR products were mixed with 100 µL probe solution (5×SSC, 0.3% Tween 20, 10 µM probe, and 50 µM competitive probe). The probes were labelled with digoxigenin at the 5’ end. To measure the methylated cytosine, the single nucleotide sequence complementary to the methylated sequence was used as a probe, and the single nucleotide sequence complementary to the non-methylated sequence was used as a competitive probe. To measure the non-methylated cytosine, the single nucleotide sequence complementary to the non-methylated sequence was used as a probe，and the single nucleotide sequence complementary to the methylated sequence was used as a competitive probe. The single nucleotide sequence complementary to the methylated sequences or non-methylated sequences are listed in Table S1. The solution was heated at 98°C for 10 min, and then immediately chilled on iced water for 10 min. Next, the solution was transferred to the well of a streptavidin coated plate, hybridized at 50°C for 2 h. After hybridization, the solution was discarded and the plate was washed twice with 300 µL PBST buffer. The hybridized digoxygenin-labeled probe was combined with an anti-digoxygenin IgG Fab fragment conjugated with alkaline phosphatase (Roche) in PBST for 30 min at room temperature, washed twice with PBST at room temperature, and detected by a colorimetric reaction with p-nitrophenyl phosphate (Thermo Fisher Scientific). Color density was measured at 405 nm by iMark Microplate Reader (Bio-Rad) and analyzed by Microplate Manager 6.0 software (Bio-Rad). All determination was performed in triplicate, and an average value was calculated for each set.

**Supplemental Table s1**

| Supplemental Table 1 Primer sequence | |
| --- | --- |
| Primers for Cloning of S22-SCR/SP11 | |
| SP11-1 | 5'-ATGAAATCTGCTATTTATGCTTTATTATG-3' |
| SP11-F1 | 5'-ATGAAATCNGNTNTTTATGCTTTATTATG-3' |
| SP11-1Fa | 5'-ATGAAATCNGNNNTTTATGCNTTATTATGTTTC-3' |
| SP11-1F8 | 5'-ATGCTTTATTATGTTTCATATTCNTCC-3' |
| SP11-2 | 5'-TTCATATTCATCGTTTCAAGTC-3' |
| SP11-F2 | 5'-TTCATATTCATCGTTTCAAGTC-3' |
| SP11-2Fa | 5'-TTCATATTCCATCNTTTNANGNCNTNNTCA-3' |
| Not1-(dT)18 | 5'-AACTGGAAGAATTCGCGGCCGCAGGAA T_18_-3' |
| RT1-long | 5'-ACTGGAAGAATTCGCGGCCGCAGGA-3' |
| SCR-22-IPCR-F | 5'-CAGAACACATCTCTGGTATTGGCAC-3' |
| SCR-22-IPCR-R | 5'-CAAGAGAAGACCGCTTTAATTGCG-3' |
| SCR-22-IPCR-NEST-F | 5'-TGCTTCCACTTCTTGAAAATGA-3' |
| SCR-22-IPCR-NEST-R | 5'-CGTGACAAGTACAAAGGATGTGTGA-3' |
| Primers for Tapetum isloating | |
| RT-SCR-60-F | 5'-CTTATGTTTCATATTTTTGATTTTGACA-3' |
| RT-SCR-60-R | 5'-GTTGATCCATTACTATTTGTTGGAA-3' |
| Primers for RT-PCR | |
| SCR-22-RT-F | 5'-GTGCCAATACCAGAGATGTGTTCTG-3' |
| SCR-22-RT-R | 5'-CGTGACAAGTACAAAGGATGTGTGA-3' |
| SCR-8-RT-F | 5'-CCACTTTAGAAGAAGAGAAATGCAA-3' |
| SCR-8-RT-R | 5'-TGCTAACACGATTTACAGTCACAAG-3' |
| SCR-36-RT-F | 5'-GAGTCCAAAGCTGCGTAACC-3' |
| SCR-36-RT-R | 5'-CGATGGCCTTCATGAATTTT-3' |
| SCR-44-RT-F | 5'-AGAGGATCATGCTTAAACTCCACGA-3' |
| SCR-44-RT-R | 5'-ACAGTAGCAATTAATCCTCCCACGA-3' |
| SCR-60-RT-F | 5'-CTATCCGAGTCCTATCTCAGGAAG-3' |
| SCR-60-RT-R | 5'-TCTGTCCCTCAACTTCATAGTGTTT-3' |
| SCR-40-RT-F | 5'-TCACAGGAACATGCGTAAATTCAGT-3' |
| SCR-40-RT-R | 5'-CAGTAGCAAGTAATCCTCCCACGAT-3' |
| SCR-29-RT-F | 5'-GTTCAAGCACTAGATGTGGGAGCTA-3' |
| SCR-29-RT-R | 5'-TGCTCTTGGTATTTAAGCATGTTCC-3' |
| Aactin-F | 5'-GCTGAGGGAAGCAAGAATGGAACC-3' |
| Aactin-R | 5'-ATCAGGAAGGACTTGTACGGTAAC-3' |
| Primers for bisulphite sequencing | |
| SCR-22-5' REGION-BIS-F | 5'-AAAAAAGATTAYGTTTTAAAGATTGAAG-3' |
| SCR-22-5' REGION-BIS-R | 5'-CCACTTCTTRTTAATTATATCAATAATC-3' |
| SCR-22-CDS-BIS-F | 5'-TTGATTATTGATATAATTAAYAAGAAGTGG-3' |
| SCR-22-CDS-BIS-R | 5'-AARAACACCTTRAAACTTCAAATATA-3' |
| SCR-44-BIS-F | 5'-GTTATTTTGTTATTTGGTTATTATG-3' |
| SCR-44-BIS-R | 5'-TTCGATTTAAACATTATCCTCTAA-3' |
| SCR-60-BIS-F | 5'-AGGAAAAGAAGAATTGGATGAGGAAGTTA-3' |
| SCR-60-BIS-R | 5'-CTCCCACATCTARTRCTTTAAATTAACAAA-3' |
| SCR-40-BIS-F | 5'-TTTATTAATTAAAATTTAAAGTGTATTT-3' |
| SCR-40-BIS-R | 5'-AATCCTAAATCCTCAACAAAAAAAA-3' |
| SCR-29-BIS-F | 5'-TGTGAAATTATTTTTAAAATGTTATTTTGT-3' |
| SCR-29-BIS-R | 5'-AAACAATTCCTAACTCCCACATCTA-3' |
| Primers for Stem-loop RT-PCR | |
| RT | 5'-GTCGTATCCAGTGCAGGGTCCGAGGTATTACGCACTGGATACGACTGTAAC-3' |
| SMI-RT | 5'-GAGACGATGTTTACGTGTAAA-3' |
| universal RT | 5'-GTGCAGGGTCCGAGGTATTC-3' |
| U6-F | 5'-TTGGAACGATACAGAGAAGATTAGCA-3' |
| U6-R | 5'-ATTTCTCGATTTATGCGTGTCATC-3' |
| Primers for precursors Smi-22 detection | |
| precursors Smi22-RT-F | 5'-TGTGACTCTTTTACACATGAAGCA-3' |
| precursors Smi22-RT-R | 5'-TTGTAACTATTTTACACGTAAACATA-3' |
| Probes for DNA Methylation State Detection by PCR-ELLSIA | |
| SCR-44-loci 1-Meth | 5'-CCTATTTCACACGTAAACATT-3' |
| SCR-44-loci 1-non-Meth | 5'-CCTATTTCACACATAAACATT-3' |
| SCR-44-loci 2-Meth | 5'-CACTTAATTGCCTATTTCACAC-3' |
| SCR-44-loci 2-non-Meth | 5'-CACTTAATTACCTATTTCACAC-3' |
| SCR-60-loci 1-Meth | 5'-CCTATTTTACACGTAAACAATTC-3' |
| SCR-60-loci 1-non-Meth | 5'-CCTATTTTACACATAAACAATTC-3' |
| SCR-60-loci 2-Meth | 5'-CACTTAATTGCCTATTTTACAC-3' |
| SCR-60-loci 2-non-Meth | 5'-CACTTAATTACCTATTTTACAC-3' |
| SCR-40-loci 1-Meth | 5'-CCTATTTCACACGTAAACATT-3' |
| SCR-40-loci 1-non-Meth | 5'-CCTATTTCACACATAAACATT-3' |
| SCR-40-loci 2-Meth | 5'-CACTTAATTGCCTATTTCACAC-3' |
| SCR-40-loci 2-non-Meth | 5'-CACTTAATTACCTATTTCACAC-3' |
| SCR-29-loci 1-Meth | 5'-CCTATTTCACACGTAAACATT-3' |
| SCR-29-loci 1-non-Meth | 5'-CCTATTTCACACATAAACATT-3' |
| SCR-29-loci 2-Meth | 5'-CACTTAATTGCCTATTTCACAC-3' |
| SCR-29-loci 2-non-Meth | 5'-CACTTAATTACCTATTTCACAC-3' |
| Primers for linkage analysis | |
| SCR-22-F | 5'-AATTGCGGCTGCACAGATTC-3' |
| SCR-22-R | 5'-TCGCAAGTCTTTCTCGAGGT-3' |
| SRK-22-F | 5'-GGCCAACAGAGATAGCCCTC-3' |
| SRK-22-R | 5'-GAACTCAGGCATTCCGCTCT-3' |
| SRK-60-F | 5'-TCATCCTGCCCTTTCGACCT-3' |
| SRK-60-R | 5'-GCGTAGGTTTTCTGGGAGACT-3' |

**
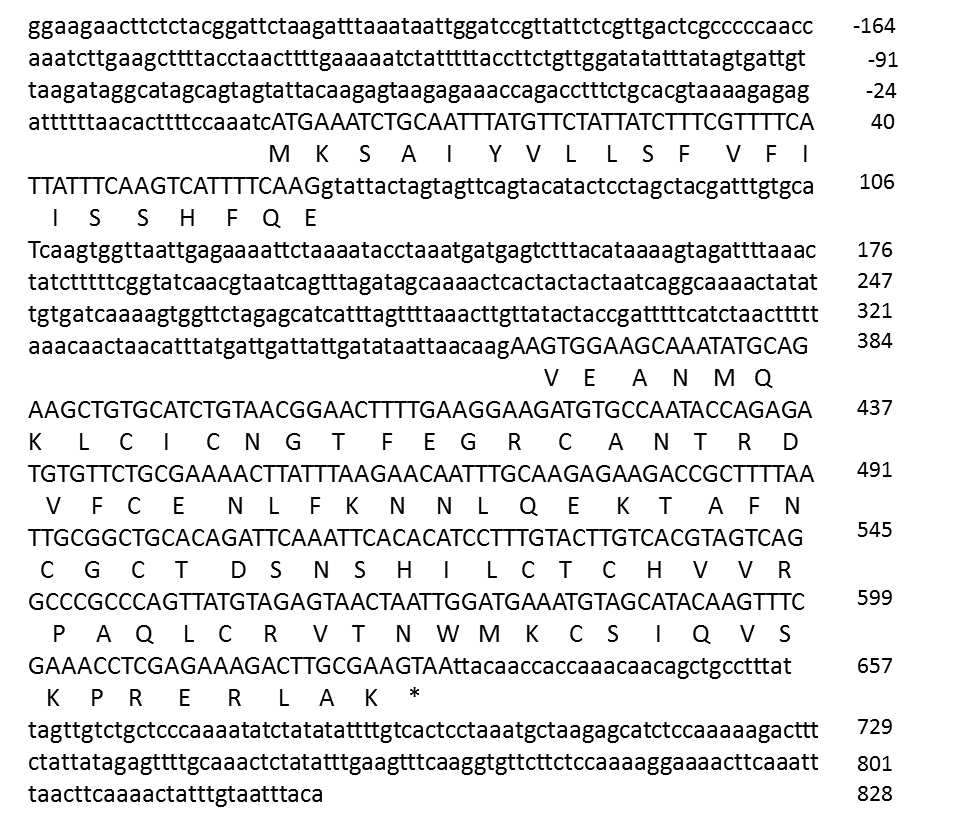
**Figure S1. Nucleotide and deduced amino acid sequences of *SCR-22*. The noncoding regions are shown in lower-case letters and coding regions are shown in upper-case letters. TAA with an asterisk is the stop codon.


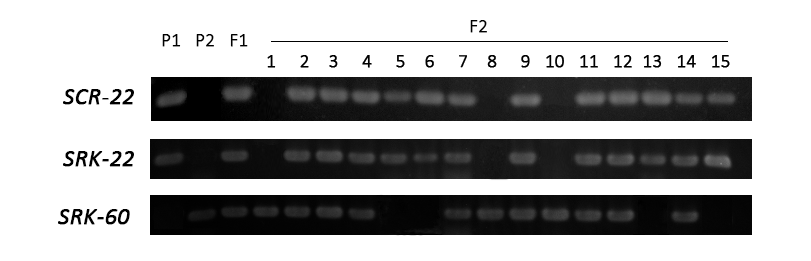
Figure S2. Linkage analysis of SCR-22 and SRK-22. The *S-22* and *S-60* homozygotes were used as parents to produce F1 generation. The F1 generation was self-pollinated to produce F2 generation.


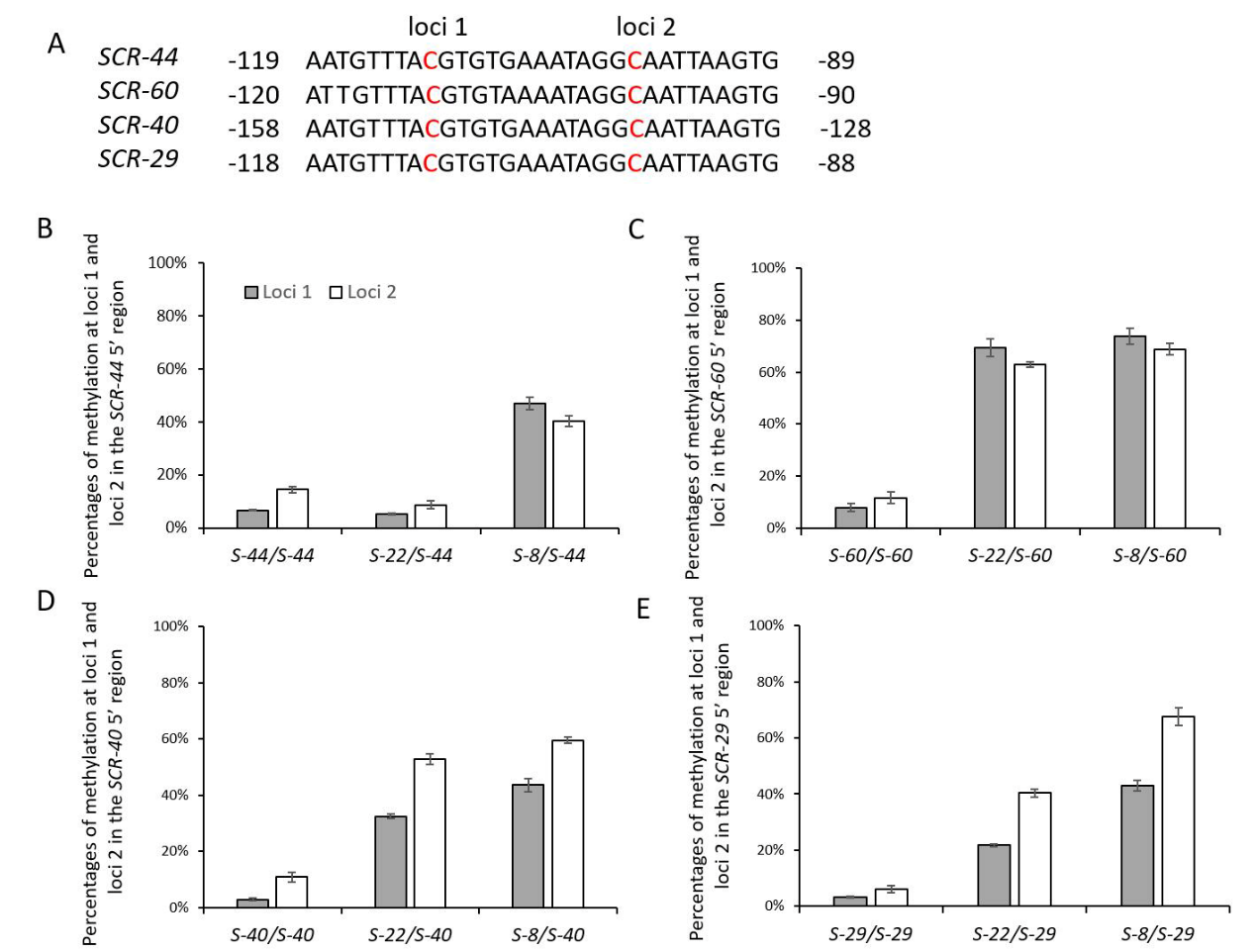


Figure S3. DNA methylation state of the cytosine in the region of class-II *SCR/SP11* alleles homologous to *Smi*. The cytosine in the sequence of *SCR-44*, *SCR-60*, *SCR-40* and *SCR-29* homologues to *Smi* were named as locus 1 and locus 2 (A). The percentages of methylation state at locus 1 and locus 2 of *SCR-44*, *SCR-60*, *SCR-40* and *SCR-29* are shown (B, C, D, and E). In this analysis, methylation levels of cytosine at locus 1 and locus 2 in the promoter region were measured by SNP detected method using PCR-ELISA[^1^](#_ENREF_1), as described in Supplemental Method.


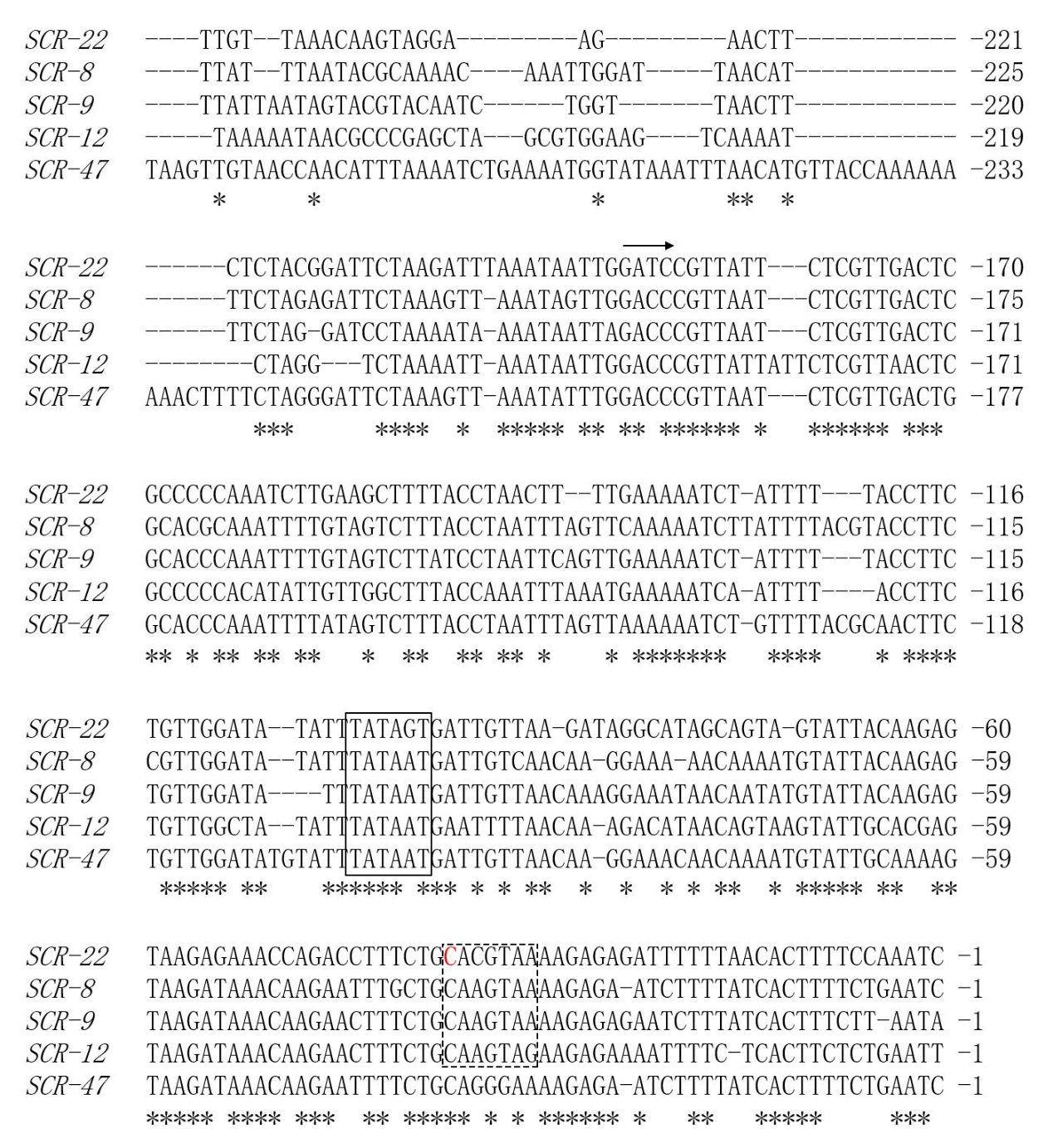


Figure S4. Alignment of the promoter sequence of five class-I *SCR/SP11* alleles. Asterisks indicate conserved DNA sequence. The red letter indicates hypermethylation. The solid line box represents the putative TATA box. The dotted line box represents the putative NAC transcription factors core binding sequence. The arrow indicates the minimal promoter region for expression in the tapetum assigned by Shiba et al.[^2^](#_ENREF_2)

**Supplemental Reference**

1 Tonosaki, K., Kudo, J., Kitashiba, H. & Nishio, T. Allele-specific hybridization using streptavidin-coated magnetic beads for species identification, S genotyping, and SNP analysis in plants. *Mol. Breed.* **31**, 419-428, doi:10.1007/s11032-012-9799-3 (2013).

2 Shiba, H. *et al.* A pollen coat protein, SP11/SCR, determines the pollen *S*-specificity in the self-incompatibility of *Brassica* species. *Plant Physiol.* **125**, 2095-2103, doi:DOI 10.1104/pp.125.4.2095 (2001).
